# Synapses-associated research in Parkinson’s disease: an explored trends analysis

**DOI:** 10.3389/fnagi.2025.1537119

**Published:** 2025-04-02

**Authors:** Yan-Jun Chen, Ming-Rong Xie, Sheng-Qiang Zhou, Fang Liu

**Affiliations:** ^1^Graduate School of Hunan University of Chinese Medicine, Changsha, China; ^2^National TCM Master Liu Zuyi Inheritance Studio, The Affiliated Hospital of Hunan Academy of Chinese Medicine, Changsha, China; ^3^The First Clinical College of Nanjing University of Chinese Medicine, Nanjing, China

**Keywords:** Parkinson’s disease, synapses, synaptic plasticity, neuroinflammation, alpha-synuclein

## Abstract

**Background:**

The pathological features of Parkinson’s disease (PD) include the formation of Lewy bodies composed mainly of aggregated alpha-synuclein (*α*-Syn) and extensive neurodegeneration. Synaptic dysfunction is a key factor contributing to disease progression among the various cellular and molecular mechanisms of PD. This study aims to analyze the research hotspots, frontier trends, and future directions of PD and synapses.

**Method:**

Relevant publications were obtained using the Web of Science database. Software CiteSpace, VOSviewer, and bibliometrix were used for visualization and quantitative analysis.

**Results:**

A total of 3,823 publications were included for analysis, and the number of publications related to the research topic showed an increasing trend from 2001 to 2024. North America, Asia, and Europe were the main research forces with high activity. The United States was the main leader in this field, followed by China and Italy. Emory University was the institution with the largest number of publications. *Journal of Neuroscience* was the core journal with a large number of publications. Dr. Calabresi, Paolo was a leader in the field of research. High-frequency keywords included PD, *α*-Syn, synaptic plasticity, basal ganglia, dopamine, substantia-nigra. In recent years, neuroinflammation has been the subject of active research.

**Conclusion:**

Communication and collaboration between different countries, institutions, and authors have promoted the development of this field. The research content mainly focused on *α*-Syn, synaptic plasticity, and mouse model. Neuroinflammation may be the direction of future research.

## 1 Introduction

Parkinson’s Disease (PD), an age-related neurodegenerative disease, is characterized by the degeneration and death of dopaminergic neurons in the substantia nigra and striatum region of the midbrain, and the abnormal aggregation of alpha-synuclein (*α*-Syn) (Kalia and Lang, 2015). These pathological changes lead to a range of clinical manifestations, including motor symptoms such as resting tremor, muscle rigidity, and bradykinesia, as well as non-motor symptoms such as sleep disorders, bowel dysfunction, and cognitive impairment (Bloem et al., 2021). These complex and diverse symptoms seriously impair the quality of life of patients and impose a heavy burden on society and families. Although there have been some breakthroughs and progress in PD research, unfortunately, there is still a lack of treatment that can effectively reverse or prevent the progression of PD. Therefore, in-depth exploration of the pathogenesis of PD has become particularly urgent, which is of great significance to promote the development of new treatment strategies to delay or curb the deterioration of the disease.

Synapses are the sites where neurons contact each other and transmit information. In the brain, synapses allow neurons to transmit electrical and chemical signals by releasing and receiving neurotransmitters, thereby coordinating and controlling various physiological and behavioral processes, which are the basis for the brain to perform multiple complex cognitive activities (Nadim and Bucher, 2014). Synaptic function is essential for the maintenance of normal brain functions, including memory (Mayford et al., 2012), perception (Froemke et al., 2013), and motor control (Hwang et al., 2022). Functional abnormality of synapses has been associated with neurological diseases, including PD (Dejanovic et al., 2024).

Studies showed that synaptic dysfunction plays a key role in the onset and progression of PD (Picconi et al., 2012; Bellucci et al., 2016). The degeneration and death of nigral dopaminergic neurons result in a decline in dopamine levels, disrupting the normal synaptic transmission between the cerebral cortex and striatum (Tritsch and Sabatini, 2012). This disruption impairs the proper functioning of the motor system and may lead to non-motor symptoms. Additionally, significant changes occur at both presynaptic and postsynaptic membranes in PD patients, including abnormal accumulation of synaptic proteins (Pagano et al., 2019), decreased neurotransmitter release (Ekman et al., 2012), and functional impairments of postsynaptic receptors (Ballanger et al., 2012). These changes exacerbate neuronal death and synaptic dysfunction, creating a vicious cycle. Exploring the pathological role of synapses in PD can help elucidate the disease’s underlying mechanisms and develop novel treatment strategies.

Bibliometrics is a research method based on statistics and bibliographic principles, which is used to quantitatively analyze and evaluate the output, dissemination, and influence of literature (Ninkov et al., 2022). The unique advantages of bibliometrics are as follows: (i) Large-scale data analysis: bibliometrics can process massive literature data and reveal hidden patterns and trends. (ii) Objectivity: analysis based on quantitative indicators (e.g., number of citations, co-occurrence analysis) reduces subjective bias. (iii) Visual presentation: bibliometrics can visually show the structure and evolution of the research field through tools such as knowledge graphs. (iv) Prospective analysis: bibliometrics can provide directional suggestions for future research by identifying emerging topics and highly cited literature. Systematic reviews typically focus on qualitative or quantitative comprehensive analyses of specific research questions, rely on subjective screening and assessment by the investigator, and are applicable to answer specific clinical or scientific questions. Besides, bibliometrics pays more attention to the macro analysis of literature data, which can reveal the overall development context, research hotspots, and collaboration networks in a certain field. In past decades, synapses-associated research in PD has been widely conducted, with numerous publications. This study used the bibliometrics approach to visually analyze the literature related to PD and synapses, aiming to reveal collaboration networks, the contribution of different research results, hotspots, frontiers, and development trends.

## 2 Methods

### 2.1 Data retrieval

Web of Science (WoS) is a comprehensive academic information library with powerful functions and rich content. It can improve the efficiency of literature retrieval, assess the influence of literature, and reveal the research hotspots and trends in the subject field (Zhang et al., 2023). The retrieved data were obtained from the WoS core collection. The search formula was ((((TS = (synapses)) OR TS = (synapsis)) OR TS = (synapse)) OR TS = (synaptic)) AND TS = (Parkinson’s Disease). The search time was set before October 31, 2024. The language was English, and the literature type was review and article. Two researchers analyzed the retrieved data and excluded publications that were not relevant to the topic. Finally, 3,823 publications were obtained for analysis.

### 2.2 Data analysis

The collected data were analyzed using CiteSpace, VOSviewer, and bibliometrix. CiteSpace is mainly used to analyze the development trends and hotspots in the research field, which helps researchers sort out literature and research planning (Chen, 2004). VOSviewer can conduct a multi-dimensional bibliometric analysis of authors, keywords, journals, and references, which has a powerful visualization effect (van Eck and Waltman, 2010). Bibliometrix is a scientific bibliometric software based on R language, which can conduct statistical analysis of related scientific literature index, construct data matrix, and visualize processing (Aria and Cuccurullo, 2017).

## 3 Results

### 3.1 Publication growth trend

**Figure 1:**
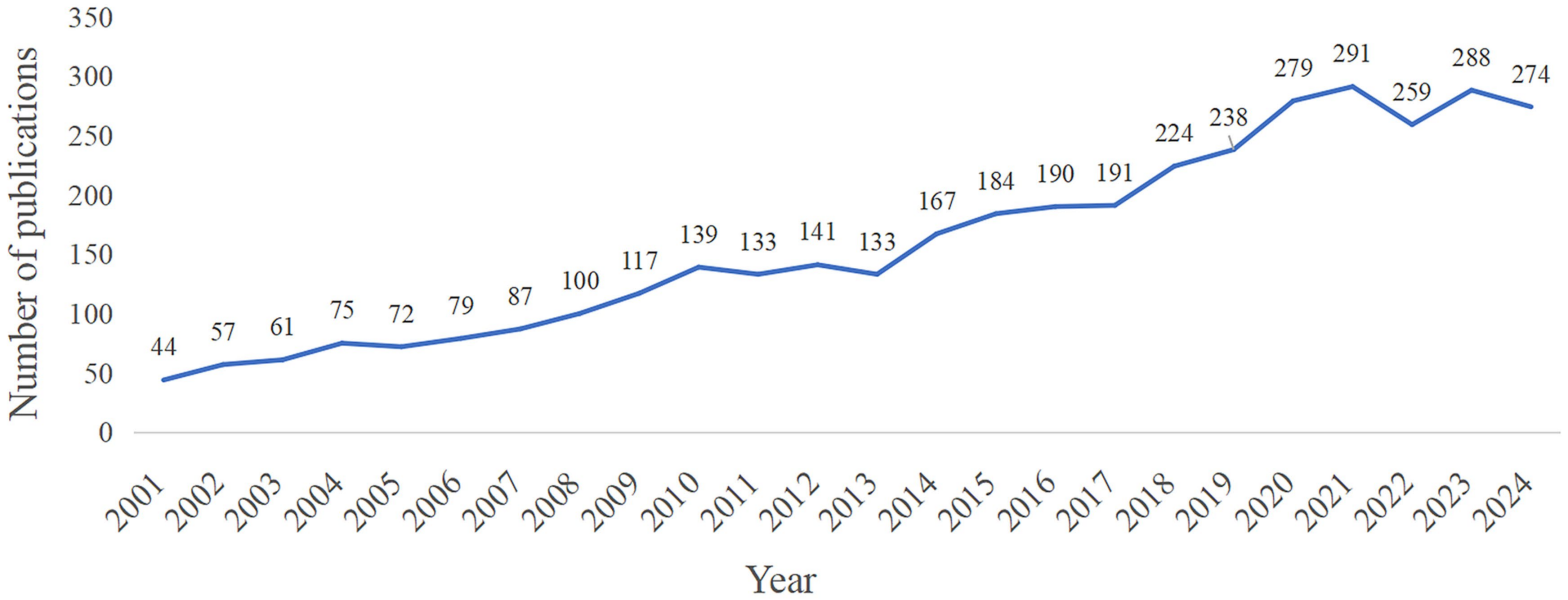
Trends in publications related to PD and synapses from 2001 to 2024.

The annual number of publications can intuitively show the heat and development trend of the research field in a period. From 2001 to 2024, a total of 3,823 publications on synapses and PD were published, and the number of publications showed an upward trend (Figure 1).

### 3.2 Geographic distribution and country

**Figure 2:**
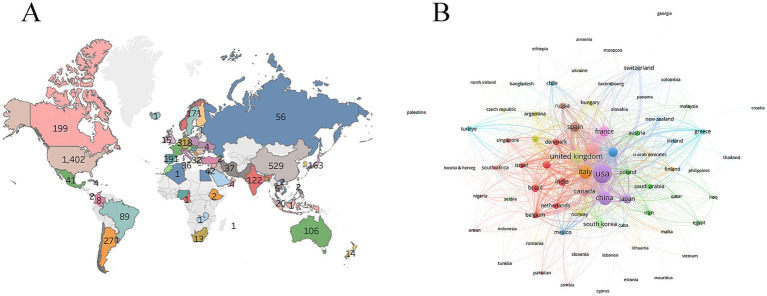
Analysis of countries. **(A)** World geographic distribution of publications. **(B)** Collaboration between countries.

The national geographic map showed that North America, Asia, and Europe were the main research forces with high activity (Figure 2A). The top three countries with the largest number of publications were the United States (1,402 publications), China (529 publications), and Italy (440 publications) (Table 1). The United States was the main leader in this field, collaborating closely with Italy, the United Kingdom, and China (Figure 2B).

**Table 1:** The top 10 countries.

Country	Number of publications	Citations
USA	1,402	108,846
China	529	14,662
Italy	440	25,680
The United Kingdom	436	29,682
Germany	318	17,597
France	236	15,212
Canada	199	11,108
Spain	191	9,947
Sweden	171	11,237
Japan	163	7,347

### 3.3 Research institutions

**Figure 3:**
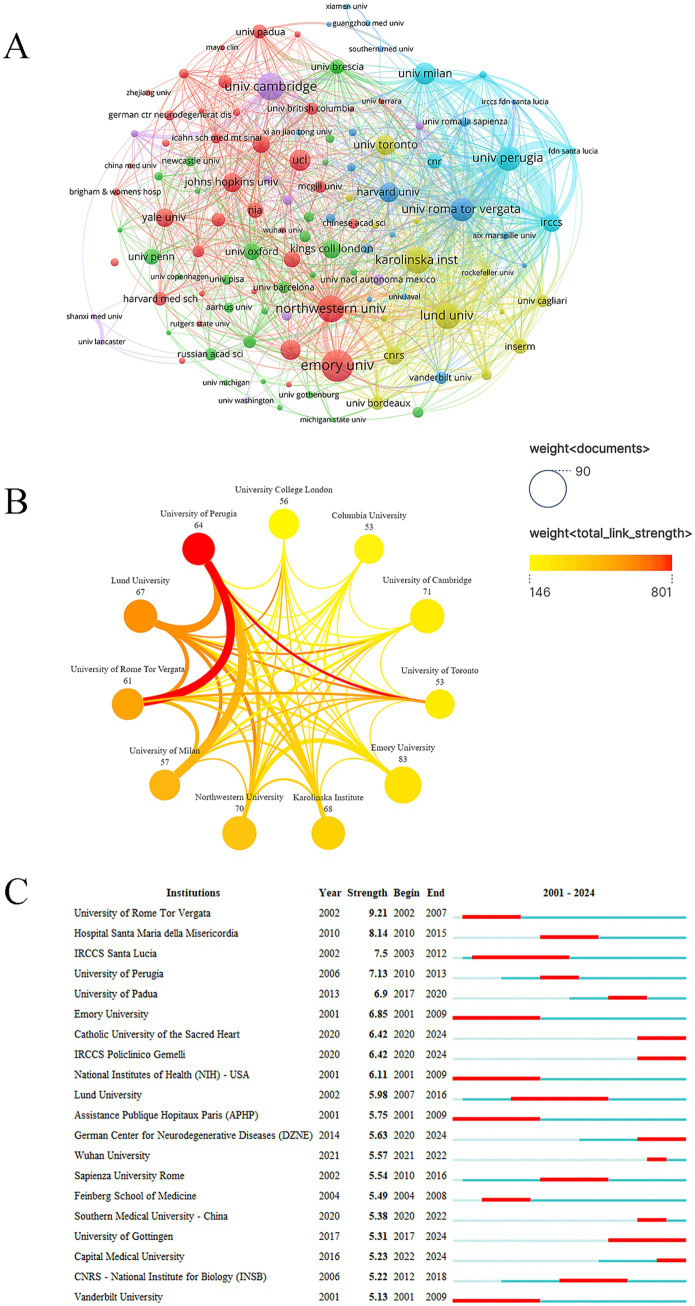
Analysis of institutions. **(A)** Collaboration between institutions. **(B)** Collaboration between the top ten institutions. **(C)** Institutions with the strongest citation bursts.

The institutional collaboration map can analyze the collaboration between different research institutions and assess the research impact. The research institution with the most publications was Emory University (83 publications), followed by the University of Cambridge (71 publications) and Northwestern University (70 publications) (Figure 3A; Table 2). Among the top ten institutions with the largest publications, the University of Perugia and the University of Rome Tor Vergata had the closest collaboration (Figure 3B). Institution burst refers to a significant increase in the number of publications or a concentrated emergence of research outcomes during a specific time. Among the top 20 research institutions with the strongest bursts, Catholic University of the Sacred Heart, IRCCS Policlinico Gemelli, German Center for Neurodegenerative Diseases, University of Gottingen, and Capital Medical University were the emerging burst forces in recent years (Figure 3C).

**Table 2:** The top 10 institutions with the most publications related to the research topic.

Rank	Institution	Documents	Citations	Average number of citations
1	Emory University	83	4,180	50.36
2	University of Cambridge	71	5,443	76.66
3	Northwestern University	70	6,970	99.57
4	Karolinska Institute	68	4,021	59.13
5	Lund University	67	6,501	97.03
6	University of Perugia	64	4,418	69.03
7	University of Rome Tor Vergata	61	5,032	82.49
8	University of Milan	57	3,273	57.42
9	University College London	56	3,596	64.21
10	University of Toronto	53	2,692	50.79
11	Columbia University	53	2,574	48.57

### 3.4 Journals and co-cited journals

**Figure 4:**
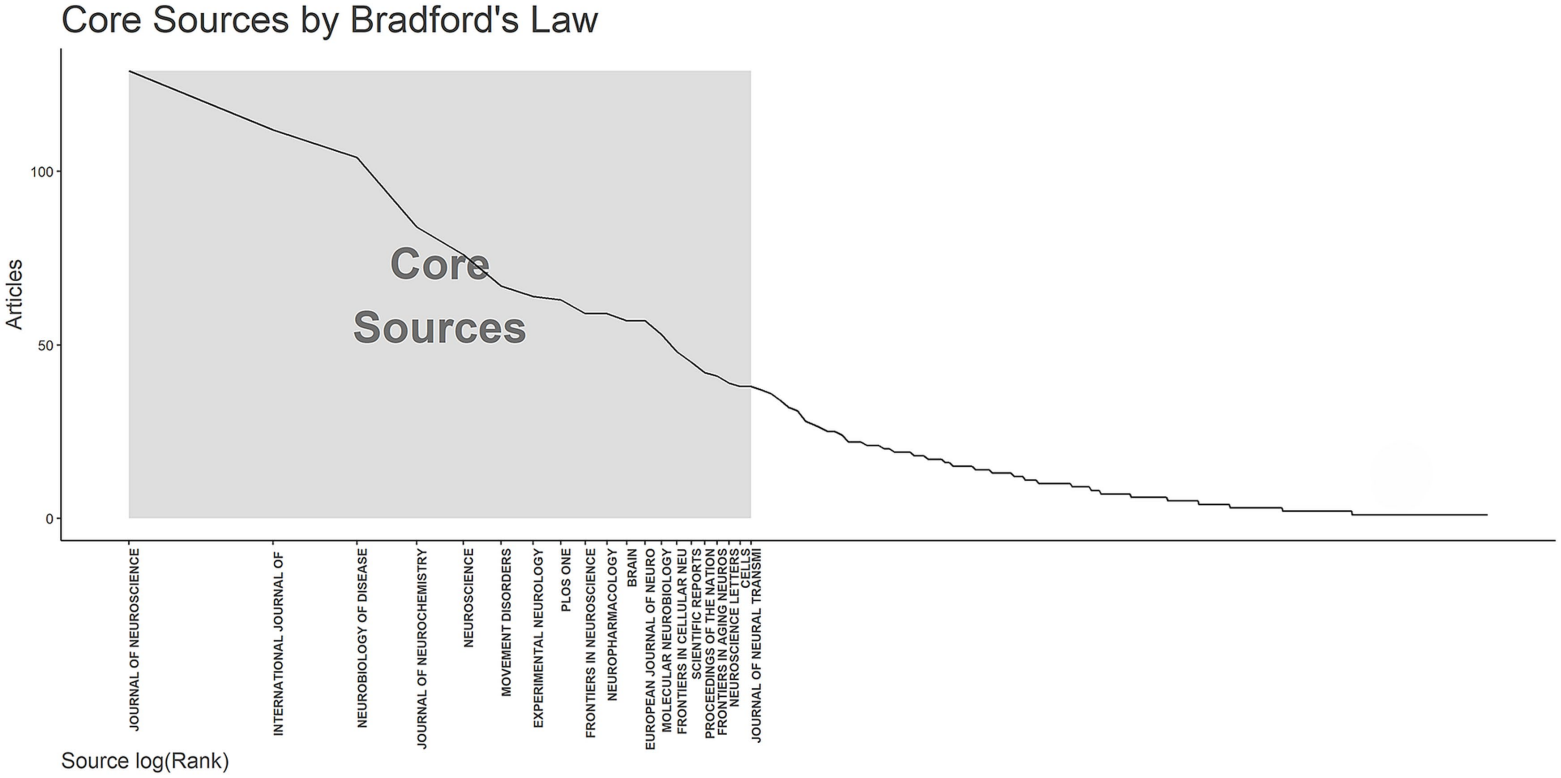
Analysis of core journals.

Journal analysis mainly evaluates the quantity, quality, citation, and other dimensions of academic journals, which helps to understand the academic level, influence, and development trend of journals. According to Bradford’s law (Bradford, 1934), 20 core journals relevant to the research topic were identified (Figure 4). The top three journals with the largest number of publications were *Journal of Neuroscience* (129 publications), *International Journal of Molecular Sciences* (112 publications), and *Neurobiology of Disease* (104 publications) (Table 3). Co-cited journal analysis is used to study the interaction and citation relationship between different journals, which can reveal the connection between different research fields, the intersection between disciplines, and research dynamics. The top three co-cited journals were *Journal of Neuroscience* (20,556 citations), *Proceedings of the National Academy of Sciences of the United States of America* (10,989 citations), and *Journal of Biological Chemistry* (9,528 citations) (Table 4).

**Table 3:** The top 10 journals.

Rank	Source	Documents	Citations	Average number of citations	IF	JCR
1	Journal of Neuroscience	129	14,322	111.02	4.4	Q1
2	International Journal of Molecular Sciences	112	3,497	31.22	4.9	Q1
3	Neurobiology of Disease	104	4,301	41.26	5.1	Q1
4	Journal of Neurochemistry	84	5,702	67.88	4.2	Q2
5	Neuroscience	76	3,687	48.51	2.9	Q2
6	Movement Disorders	67	2,970	44.33	7.4	Q1
7	Experimental Neurology	64	2,615	40.86	4.6	Q1
8	PLosOne	63	2,275	36.11	2.9	Q1
9	Frontiers in Neuroscience	59	2,923	49.54	3.2	Q2
10	Neuropharmacology	59	3,027	51.31	4.6	Q1

IF, Impact Factor; JCR, Journal Citation Reports.

**Table 4:** The top 10 co-cited journals.

Rank	Source	Citations	IF	JCR
1	Journal of Neuroscience	20,556	4.4	Q1
2	Proceedings of the National Academy of Sciences of the United States of America	10,989	9.4	Q1
3	Journal of Biological Chemistry	9,582	4	Q2
4	Neuron	8,909	14.7	Q1
5	Journal of Neurochemistry	7,503	4.2	Q2
6	Nature	7,255	50.5	Q1
7	Science	6,775	44.8	Q1
8	Neuroscience	6,056	2.9	Q2
9	Movement Disorders	5,934	7.4	Q1
10	Brain Research	5,752	2.7	Q3

IF, Impact Factor; JCR, Journal Citation Reports.

### 3.5 Co-cited references

Co-cited reference analysis can quickly locate highly cited or high-impact literature related to the topic, thereby improving the pertinence and efficiency of literature collection. The most cited literature was “*Alpha-synuclein in Lewy bodies* (Spillantini et al., 1997)” (383 citations), followed by “*Mutation in the alpha-synuclein gene identified in families with Parkinson’s disease* (Polymeropoulos et al., 1997)” (364 citations) and “*Staging of brain pathology related to sporadic Parkinson’s disease* (Braak et al., 2003)” (268 citations) (Table 5).

**Table 5:** The top 10 co-cited references.

Rank	Title	Type	Citation times	Year	Journal	IF	JCR
1	Alpha-synuclein in Lewy bodies.	Article	383	1997	Nature	50.5	Q1
2	Mutation in the alpha-synuclein gene identified in families with Parkinson’s disease.	Article	364	1997	Science	44.8	Q1
3	Staging of brain pathology related to sporadic Parkinson’s disease	Review	268	2003	Neurobiology of Aging	3.7	Q2
4	α-synuclein locus triplication causes Parkinson’s disease	Article	237	2003	Science	44.8	Q1
5	α-Synuclein Promotes SNARE-Complex Assembly *in Vivo* and *in Vitro*	Article	232	2010	Science	44.8	Q1
6	Loss of bidirectional striatal synaptic plasticity in L-DOPA-induced dyskinesia	Article	232	2003	Nature Neuroscience	21.3	Q1
7	Ala30Pro mutation in the gene encoding alpha-synuclein in Parkinson’s disease.	Article	222	1998	Nature Genetics	31.7	Q1
8	Increased Expression of α-Synuclein Reduces Neurotransmitter Release by Inhibiting Synaptic Vesicle Reclustering after Endocytosis	Article	217	2010	Neuron	14.7	Q1
9	Mice lacking alpha-synuclein display functional deficits in the nigrostriatal dopamine system.	Article	211	2000	Neuron	14.7	Q1
10	The functional anatomy of basal ganglia disorders.	Article	209	1989	Trends in Neurosciences	14.6	Q1

**Figure 5:**
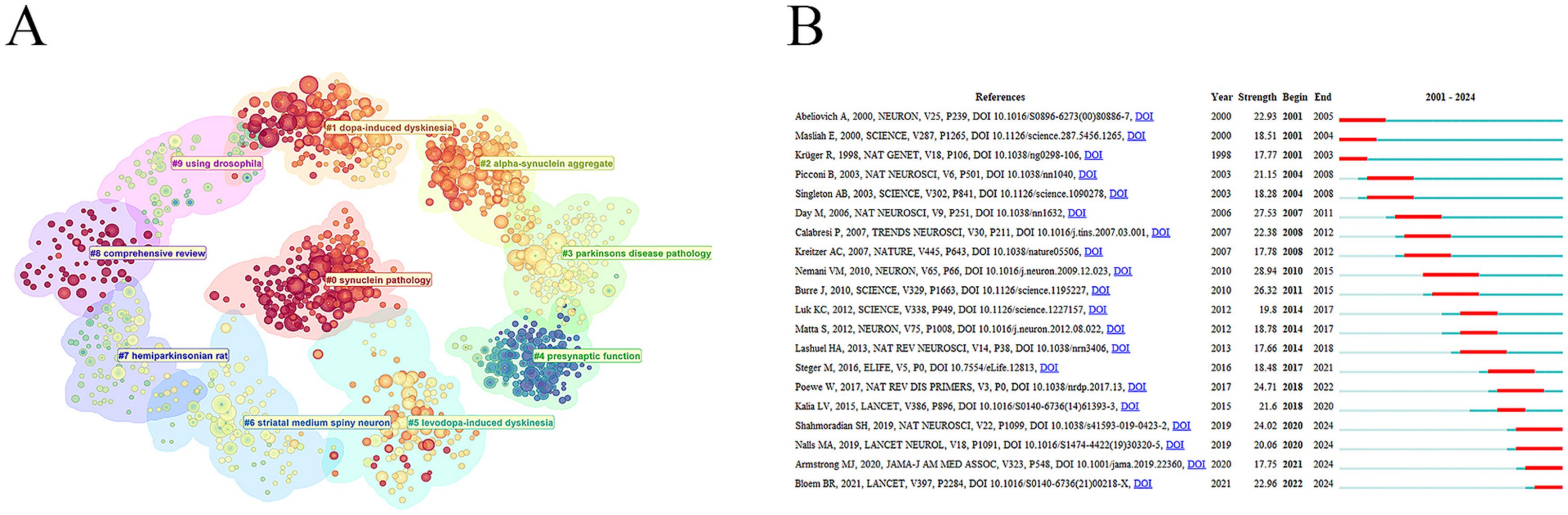
Analysis of references. **(A)** Reference cluster analysis. **(B)** References with the strongest citation bursts.

In the cluster analysis of co-cited references, each cluster represents a research topic or hotspot direction. The color of the cluster from blue to yellow to deep red reflected the change of research hotspots in different periods (Figure 5A). “presynaptic junction” received more attention in the past, but in recent years, the research direction has shifted to “synuclein pathology,” “dopa-induced dyskinesia,” and “alpha-synuclein aggregate”.

Reference burst refers to the large number of citations of literature in a certain period, reflecting the significant increase in the attention and influence of this research in the academic community. Papers “*Lewy pathology in Parkinson’s disease consists of crowded organelles and lipid membranes* (Shahmoradian et al., 2019),” “*Identification of novel risk loci, causal insights, and heritable risk for Parkinson’s disease: a meta-analysis of genome-wide association studies* (Nalls et al., 2019),” “*Diagnosis and Treatment of Parkinson Disease: A Review* (Armstrong and Okun, 2020),” and “*Parkinson’s disease* (Bloem et al., 2021)” were the references with strong burst power in recent years (Figure 5B).

### 3.6 Authors and co-cited authors

**Figure 6:**
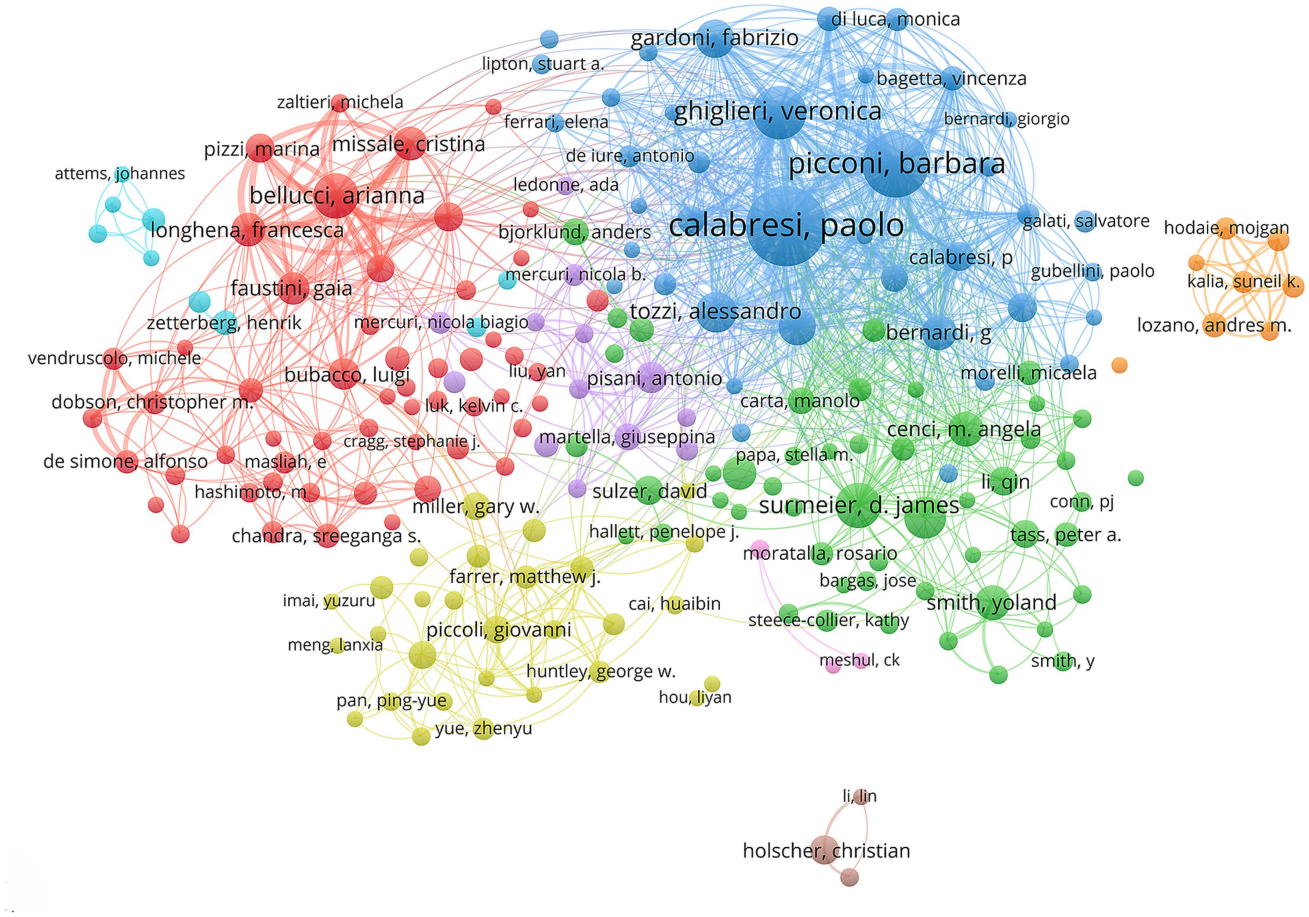
Analysis of authors.

Authors with a large number of publications are usually regarded as the core authors in the field, and their research has high attention and influence. The most prolific author was Dr. Calabresi, Paolo (60 papers), followed by Dr. Picconi Barbara (46 papers) and Dr. Ghiglieri Veronica (33 papers) (Table 6). In the author’s collaboration network graph, different colors represent different research teams (Figure 6). The blue team with the largest number of publications was led by scholars Dr. Calabresi, Paolo, and the core members included Dr. Picconi Barbara, Dr. Ghiglieri Veronica, and Dr. Fabrizio Gardoni.

**Table 6:** The top 10 authors.

Rank	Author	Documents	Citations	Average number of citations	Country	Institution
1	Dr. Calabresi, Paolo	60	4,035	67.25	Italy	Catholic University of the Sacred Heart
2	Dr. Picconi Barbara	46	3,350	72.83	Italy	IRCCS San Raffaele Pisana
3	Dr. Ghiglieri, Veronica	33	2,650	80.30	Italy	University of San Raffaele
4	Dr. Bellucci, Arianna	26	1,193	45.88	USA	National Institute of Neuroscience
5	Dr. Surmeier, Dalton James	26	3,552	136.62	USA	Northwestern University
6	Dr. Bezard, Erwan	23	987	42.91	France	University of Bordeaux
7	Dr. Tozzi, Alessandro	22	1707	77.59	Italy	University of Perugia
8	Dr. Gardoni, Fabrizio	21	888	42.29	Italy	University of Milan
9	Dr. Di Filippo, Massimiliano	20	2,331	116.55	Italy	University of Perugia
10	Dr. Bernardi, G	18	2,947	163.72	Italy	University of Rome Tor Vergata
11	Dr. Cenci, Maria Angela	18	1957	108.72	Sweden	Lund University
12	Dr. Smith, Yoland	18	866	48.11	USA	Emory University

Authors with high co-citation frequency indicate that their studies have received high attention and influence, and contribute to the development of the field. The highest co-citation was Dr. Calabresi, Paolo (1,004 citations), followed by Dr. Braak, Heiko (719 citations) and Dr. Spillantini, Maria Grazia (655 citations) (Table 7). Notably, Dr. Calabresi, Paolo was an author with significant academic influence, who had published the largest number of papers and received the most citations.

**Table 7:** The top 10 co-cited authors.

Rank	Author	Citations	Country	Institution
1	Dr. Calabresi, Paolo	1,004	Italy	Catholic University of the Sacred Heart
2	Dr. Braak, Heiko	719	German	Ulm University
3	Dr. Spillantini, Maria Grazia	655	England	University of Cambridge
4	Dr. Picconi, Barbara	527	Italy	IRCCS San Raffaele Pisana
5	Dr. Gerfen, Charles R.	406	USA	National Institutes of Health
6	Dr. Polymeropoulos, Mihael H.	387	USA	National Institutes of Health
7	Dr. Mattson, Mark P.	387	USA	National Institutes of Health
8	Dr. Surmeier, Dalton James	386	USA	Northwestern University
9	Dr. Cenci, Maria Angela	382	Sweden	Lund University
10	Dr. Smith, Yoland	350	USA	Emory University

### 3.7 Keywords

**Figure 7:**
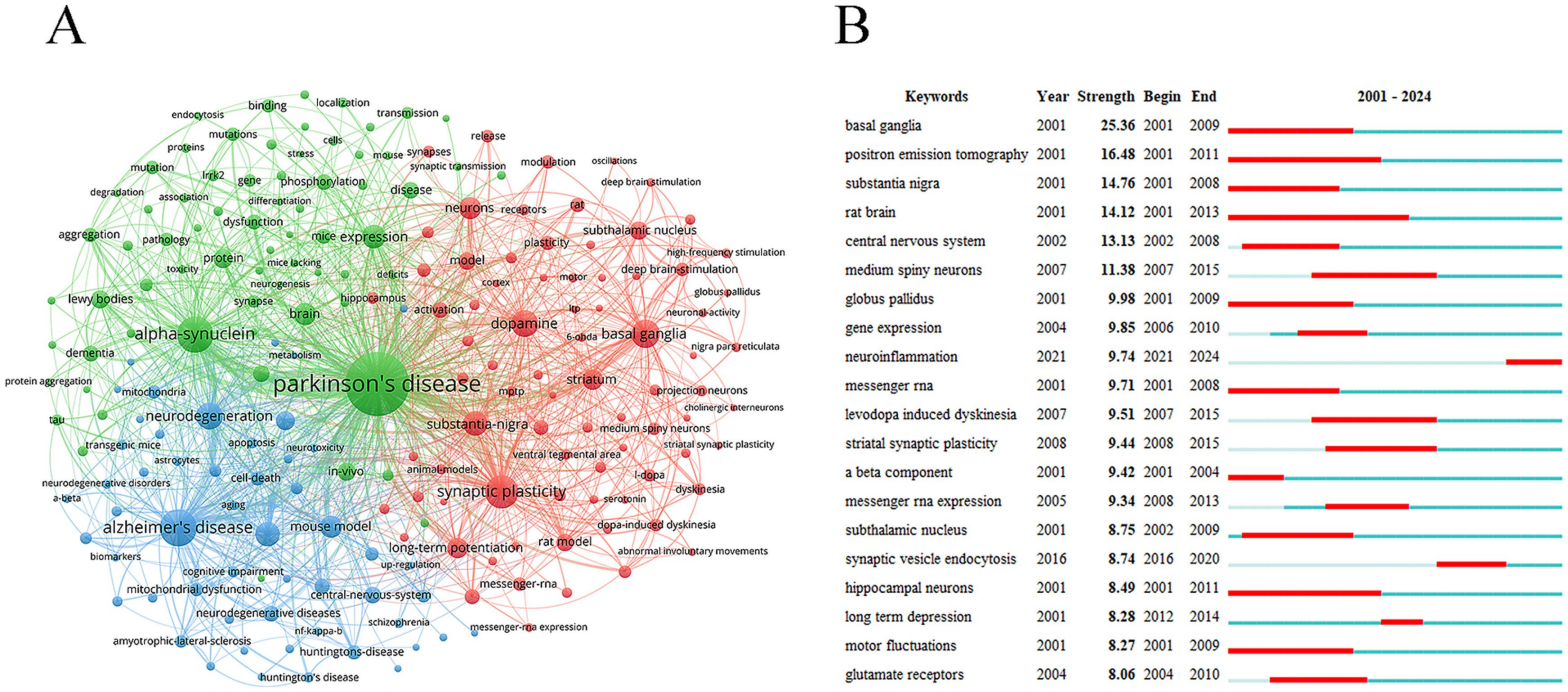
Analysis of keywords. **(A)** Keywords network diagram. **(B)** Keywords with the strongest citation bursts.

Keyword analysis can reveal the main research directions and hotspots in the research field. PD (2,138 times) *α*-Syn (722 times) synaptic plasticity (611 times) basal ganglia (463 times) dopamine (412 times) substantia-nigra (349 times) oxidative stress (339 times) and mouse model (288 times) were high-frequency keywords (Figure 7A). Keywords with the strongest citation bursts reveal current research hotspots and indicate future research directions. The keyword with the strongest burst in recent years was “neuroinflammation” (strength = 9.74) (Figure 7B).

## 4 Discussion

Relevant studies showed that synapses are closely related to the occurrence and development of PD, and synaptic dysfunction plays an important role in the pathological mechanism of PD. This study aims to explore the research hotspots and frontier trends of PD and synapses, providing new insights for treating PD.

A total of 3,823 publications were included for analysis, and the number of publications related to the research topic showed an increasing trend from 2001 to 2024. The increase in publications reflected the active level of research activities and the continuous expansion of subject areas. The National Geographic map showed that North America, Asia, and Europe were the main research forces with high activity. The United States was the main leader in this field, followed by China and Italy. It should be emphasized that the related publication output of synapses and PD only reflected the number of papers of an institution in the field of PD, which was not directly equivalent to scientific research efficiency or influence. This analysis focused on synaptic and PD-related publications and does not consider the primary publications in the field of PD research of institutions or researchers in other fields. Emory University, the University of Cambridge, and Northwestern University were the top three institutions in the number of publications on this research topic. The reason for this phenomenon may be the relatively high burden of PD in these countries due to the older population. Therefore, research institutions and scholars in these countries pay high attention to the research of PD, which has produced numerous research outcomes.

Core journals usually represent the mainstream research direction and the latest progress in a certain subject field. *Journal of Neuroscience*, *International Journal of Molecular Sciences,* and *Neurobiology of Disease* were the journals with the largest number of publications. These journals have high academic levels and influence and were located in the JCRQ1. The top three co-cited journals were *Journal of Neuroscience*, *Proceedings of the National Academy of Sciences of the United States of America*, and *Journal of Biological Chemistry*. Co-cited journal analysis can show the academic connection and mutual influence among different journals. These connections may be based on common research topics, methods, or subject areas, thus revealing scholarly relevance and disciplinary structure among journals.

Co-citation frequency is an important index to assess the importance and influence of literature. The publication titled “*Alpha-synuclein in Lewy bodies*” was the most cited, which represented the foundation of the research field and strongly promoted the in-depth research and development of the discipline. The top ten co-cited literature focused on the pathological studies of *α*-Syn. These findings improved researchers’ understanding of the specific pathogenesis of PD, especially in the physiological and pathological aspects related to synapses, and provided a new perspective for subsequent research exploration and clinical application.

The active authors with a large number of publications are usually experts in the field, and their research outcomes have important contributions to the development of the field. Notably, Dr. Calabresi, Paolo was an author with significant academic influence, who had published the largest number of papers and received the most citations. Dr. Calabresi, Paolo and his team focused on the following aspects: (i) the effect of synaptic plasticity on motor function in PD (Pisani et al., 2005; Marino et al., 2023); (ii) specific mechanisms of synaptic dysfunction (Bagetta et al., 2010; Schirinzi et al., 2016); (iii) novel neuroprotective strategies targeting synapses (Bagetta et al., 2010; Mancini et al., 2020; Ferrari et al., 2022). Their studies revealed the potential therapeutic value of synaptic plasticity in PD and provided a therapeutic target for synaptic dysfunction. These findings provided new ideas and directions for the treatment of PD.

Keyword analysis can reveal the main research directions and hotspots in the research field. PD *α*-Syn synaptic plasticity basal ganglia dopamine substantia-nigra and mouse model were high-frequency keywords. α-Syn a soluble neuronal protein is widely expressed in the presynaptic terminal. In physiological conditions α-Syn regulates the homeostasis of neuron cell membranes controls synaptic vesicle transport and regulates dopamine synthesis (Calo et al., 2016). However under pathological conditions accumulated α-Syn not only impairs the function of synaptic vesicle transport but also alters the activities of vesicular monoamine transporter 2 and dopamine transporter to impair dopamine transport and uptake (Bridi and Hirth, 2018). The abnormal aggregation and deposition of α-Syn is an essential component of the Lewy body which may lead to the degenerative death of dopaminergic neurons thereby triggering PD (Burré, 2015). Additionally genetic variants in the *SNCA* gene encoding α-Syn are associated with PD (Chen et al., 2024). Synaptic plasticity refers to the tunable property of the strength in synaptic connections. A study showed that synaptic plasticity of STN-ANT circuit is associated with motor activity in PD mice. The STN-ANT circuit may transmit the motor signals from the basal ganglia to the cingulate cortex participate in the processing of sensorimotor information and synaptic plasticity and thus play a role in the regulation of motor dysfunction in PD (Zhang et al., 2021). High-intensity aerobic exercise can ameliorate the motor function of rats with PD which may be related to increasing the expression of brain-derived neurotrophic factors to enhance the synaptic plasticity of the striatum (Marino et al., 2023). Reduced transmission efficiency at nigrostriatal synapses was observed in the heterozygous PINK1 mouse model exhibiting an “indolent” state and this change may affect the normal function of neurons (Calabresi and Ghiglieri, 2014). In Pink1 knockout mice impaired synaptic transmission function and abnormal synaptic morphology were found which were characterized by significantly reduced frequency and amplitude of spontaneous excitatory postsynaptic currents in dopaminergic neurons abnormal morphology of presynaptic and postsynaptic membranes widened synaptic cleft and decreased expression of synaptic proteins (Pearlstein et al., 2016).

Keyword burst analysis can reveal the hotspots and emerging trends in the current research field by focusing on the significant increase in keyword frequency during a certain period. This analysis helps researchers to quickly grasp the frontier of the discipline and understand the latest research directions and progress. Neuroinflammation was the keyword with the strongest burst in recent years. Abnormal aggregation of *α*-Syn can not only activate NLRP3 inflammasome in microglia and increase IL-1β production but also activate the TLR4 pathway in astrocytes thereby mediating neuroinflammation (Rannikko et al., 2015; Huang et al., 2023). Inhibition of p38 alpha can ameliorate synaptic degeneration and neuroinflammation in mice overexpressing *α*-Syn (Iba et al., 2023). Glutamate is an important central neurotransmitter which may trigger neuroinflammation by activating glial cells when its level is abnormally elevated. At the same time the dysfunction of astrocytes in the process of glutamate clearance may also lead to the accumulation of glutamate in the synaptic cleft thereby aggravating excitotoxicity in PD (Iovino et al., 2020). Neuroinflammatory response mediated by synaptic dysfunction may be a future trend and hotspot.

This study also has some limitations. First, the data collected were only from the WoS database, and literature from other databases was not included. Second, the language type was limited to English and the document type was limited to article and review, which may leave out a small number of publications. Third, bibliometric analysis mainly reveals research trends and patterns at the macro level, and future research can incorporate content analysis to improve the depth and quality of further exploration research.

## 5 Conclusion

The number of publications related to PD and synapses has gradually increased. Communication and collaboration between different countries, institutions, and authors have promoted the development of this field. The research content mainly focused on α-Syn, synaptic plasticity, and mouse model. Neuroinflammation may be the direction of future research.

## Data Availability

The original contributions presented in the study are included in the article/supplementary material, further inquiries can be directed to the corresponding authors.
